# Clinical and imaging characteristics of primary hepatic sarcomatoid carcinoma and sarcoma: a comparative study

**DOI:** 10.1186/s12885-020-07475-z

**Published:** 2020-10-09

**Authors:** Dongli Shi, Jun Sun, Liang Ma, Jing Chang, Hongjun Li

**Affiliations:** 1 Department of Diagnostic Radiology, Beijing You’an Hospital, Capital Medical University, No.8, Xi Tou Tiao, You’anmen wai, Fengtai District, Beijing, 100069 China; 2 Center of Interventional Oncology and Liver Diseases, Beijing You’an Hospital, Capital Medical University, No.8, Xi Tou Tiao, You’anmen wai, Beijing, 100069 Fengtai District China; 3 Department of pathology, Beijing You’ an Hospital, Capital Medical University, No.8, Xi Tou Tiao, You’anmen wai, Beijing, 100069 Fengtai District China

**Keywords:** Liver tumor, Sarcomatous carcinoma, Sarcoma, Computed tomography, Magnetic resonance imaging

## Abstract

**Background:**

Primary hepatic sarcomatous carcinoma (PHSC) and primary hepatic sarcoma (PHS) are rare malignancies with frequent overlap in both the clinic and radiology. No comparative study of these tumors for the restricted cases has previously been undertaken. The purpose of our study was to analyze the clinical and imaging features of PHSCs and PHSs, with an emphasis on particularities and similarities through a comparison of the two tumors.

**Methods:**

We retrospectively analyzed the clinical and imaging features of 39 patients with pathologically proven PHSCs (*n* = 23) and PHSs (*n* = 16) from four university centers over a 9-year period from 2010 to 2019. Univariate analyses were performed to determine the consistent and distinctive features.

**Results:**

The background of chronic hepatitis or cirrhosis was observed with a high frequency in both of PHSCs (73.7%) and PHSs (62.5%). Tumors with a diameter greater than 10 cm were significantly more common in PHSs than PHSCs (*p* = 0.043) and cystic masses were more detected in PHSs (*P* = 0.041). Both PHSCs and PHSs mainly presented hypovascularity (78.3% vs 81.3%). The ring hyper enhancement on the arterial phase (AP) and wash out were more frequently seen in PHSCs and the iso-hypo enhancement on the AP followed persistent or progressive enhancement was more commonly detected in PHSs (all, *p* < 0.05).

**Conclusion:**

PHSC and PHS generally present as mass lesions with hypovascularity. The ring hyper enhancement on the AP and wash out favor the diagnosis of PHSC. The large size greater than 10 cm, cystic lesion, iso-hypo persistent or progressive enhancement pattern might suggest the possibility of PHSs.

## Background

Primary hepatic sarcomatous carcinoma (PHSC) and primary hepatic sarcoma (PHS), are rare malignancies accounting for only 0.2% [1] and 1% [2] of primary malignant liver tumors, respectively. Sarcomatous carcinoma is defined as a tumor containing an intimate mixture of carcinomatous (either hepatocellular or cholangiocellular) and sarcomatous elements. Sarcomatous change in hepatocellular carcinoma (HCC) or intrahepatic cholangiocarcinoma (ICC) is defined as “sarcomatous HCC (S-HCC)” or “sarcomatous ICC (S-ICC)” in the World Health Organization (WHO) classification [3]. This entity is differentiated from a true hepatic sarcoma, such as undifferentiated embryonal sarcoma (UES), leiomyosarcoma (LS), malignant solitary fibrous tumor (SFT), epithelioid sarcoma (ES) and other interstitial tumors deriving from the liver. It should be diagnosed as sarcomatous carcinoma when the sarcomatous component is predominantly composed of spindle cells, but the epithelial cells are still morphologically, immunohistochemically, and ultrastructurally identifiable [1].

The PHSC and PHS have many overlapping features in imaging as well as clinical and pathological findings [4–6], but their treatment modalities may be different, even though the most optimal therapy still awaits further evidence, due to the dearth of available information caused by their rarity. In the group of PHSs, recent studies have suggested that a combination of surgery and pre- or post-surgical chemotherapy can substantially improve clinical outcomes of the UES. For unresectable tumors, systemic chemotherapy and local radiotherapy can be options [7]. In the case of PHSCs, surgery would be justified as the primary treatment. TACE may prove effective in prolonging the survival of patients with unresectable intrahepatic recurrences [8]. Therefore, an accurate diagnosis is crucial for determining therapeutic planning.

Clinically, these tumors are usually asymptomatic until they become significantly large by the time of diagnosis, and most tumor markers are not sensitive [8–10]. The fine needle biopsy usually failed to determine the nature of the mass due to its large size and insufficient samples. Preoperative diagnosis by imaging may prevent unwarranted diagnostic surgical procedures.

The current literature on these tumors is limited to either case reports or small case series [11–15], yet no reports comparing the two tumors were available, except a mention by Mani, H [16]. Although these tumors have frequent overlap in clinical and imaging appearances, there still exist some features that could suggest a diagnosis.

Our research aims to explore the clinical and imaging features that can aid in differentiating PHSCs from PHSs.

## Methods

### Patients

We retrospectively reviewed patients from four university centers between January 2011 and April 2019 pathologically proven to have PHSC and PHS according to the World Health Organization definition of 2000. For inclusion, none of the subjects had any prior treatment of the evaluated lesions. In the PHSC group, one patient with preoperative intervention by transcatheter arterial chemoembolization (TACE) and two patients with liver metastasis from extrahepatic origin of SC were excluded. In the PHS group, sarcomas of vascular origin including epithelioid hemangioendothelioma (*n* = 7), angiosarcoma (*n* = 8), and Kaposi sarcoma (*n* = 1) were excluded for their relative specificity in the imaging or clinical characteristics. Our study included PHSCs (*n* = 23, 11 S-HCCs, 4 S-ICCs, 1 S-HCC–CC, 7 unclassified) and PHSs (*n* = 16, 1 UES, 2 SFTs, 2 ES, 3 LSs, 8 unclassified sarcomas). Clinical materials (including demographic characteristics, laboratory data, clinical symptoms and prognosis), imaging findings and pathology results were reviewed. Approval for the study protocol was obtained from the Institutional Review Board of each hospital.

### Imaging

#### CT techniques

Twenty-one patients with PHSC and all patients with PHS were instructed to complete examinations using the Computed tomographic (CT) scanner (LightSpeed VCT 64, GE Healthcare, Waukesha, Wisconsin, USA) with the following parameters: tube voltage, 120 kV; tube current, 189–200 mA; matrix, 512 × 512 mm; and section thickness 5 mm. All patients underwent dynamic three-phase scanning including hepatic arterial phase (HAP) (25–40s), portal venous phase (PVP) (45–90s) and equilibrium phase (EP) (2-5 min) which were obtained following bolus injection of contrast agent with lopromide (Ultravist 370, Bayer Schering Pharma, Berlin, Germany) at a dose of 1.5 mL/kg and rate of 3 mL/s.

#### MRI techniques

Nine patients with PHSC and four patients with PHS were instructed to complete examinations using the 3.0 T whole-body MRI systems (Trio, Siemens Healthineers, Erlangen, Germany) with an 8-channel phased array body coil. The parameters of T1-weighted fast low angle shot sequence were mentioned as below: TR/in phase: TE, 170/2.30; out-of phase TE, 3.67 ms; matrix size, 256 × 205; flip angle, 65°. The three-dimensional volumetric interpolated breath-hold examination (3D-VIBE) sequence was obtained in advance (pre-contrast) and after the injection of contrast agent (Gd-BOPTA, MultiHance, Bracco Pharma, Italy) at a rate of 2 ml/s. The serial dynamic contrast-enhanced scans including HAP, PVP and EP were collected at the time of 25–40 s, 45–90 s and 2–5 min.

#### Image analysis

All images were retrospectively assessed by two abdominal radiologists with over seven years’ experience of hepatic imaging. In the case of disagreement in assessment of the images, the two readers were required to reassess them together.

For morphological lesion assessment, the following items were evaluated: 1. The location (right lobe, left lobe), 2. Size (> 10 cm, ≤10 cm), 3. Contour (round, lobulated or irregular), 4. Margin (sharp and indistinct), 5. Liver surface contour (retraction, smooth, bulging), 6. The presence of capsule appearance, hemorrhage, and perfusion alteration, 7. The cystic lesion (The cystic lesion was evaluated based on the predominant parts (75%) of the tumor with cystic changes without any enhancement), 8. The presence of vascular invasion, intrahepatic metastasis and extrahepatic metastasis. AP enhancement was classified according to the categorizations provided by Rimola et al. with modifications [17], 9. Non-ring enhancement include the global enhancement that hyperenhancement involving > 75% of the lesion and the nodular enhancement that hyperenhancement involving 25–75% of the lesion; ring enhancement include the peripheral enhancement that hyperenhancement involving 25–75% of the lesion and rim enhancement that rim-like hyperenhancement involving < 25% of the lesion), and iso-hypointensity/density. 10. The vascularity of the whole tumor (lesions with heterogeneous enhancement were evaluated based on the predominant parts more than half of the entire tumor), 11. Dynamic pattern of enhancement (washout, progressive or persistent enhancement).

### Statistical analysis

Continuous variables, including the age of patients and the diameter of tumors, were expressed as mean ± SD, and the differences between the PHSC and PHS groups were conducted using the independent t-test. The categorical variables were compared using Fisher’s exact test. P<0.05 was considered to indicate a statistically significant difference. All statistical analyses were performed with the software SPSS® version 23.0 (IBM, Armonk, NY, USA).

## Results

### Patient characteristics and clinical background

The PHSC cohort included 23 patients (20 men, 3 women, median age, 56 years; range, 32–77 years), and the PHS cohort consisted of 16 patients (11 men, 5 women, median age, 58 years; range, 22–75 years). The pathologically-proven diagnosis was obtained after surgical resection (15 PHSCs, 6 PHSs) or biopsy (8 PHSCs, 10 PHSs). The clinical data of the patients with PHSC and PHS are summarized in Table 1. There were no significant differences found in the tumor markers. The majority of patients in both groups were middle-aged men (87.0% vs 68.8%) with the background of liver cirrhosis (73.7% vs 62.5%). The most common complaints in PHS and PHSC were abdominal discomfort (43.8% vs 30.4%) and in PHSCs, 34.8% of these patients were detected incidentally in their routine checkup for hepatitis or another disease. Vascular invasion (56.5% vs 25.0%), intrahepatic metastasis (40.9% vs 25.0%), and extrahepatic metastasis (57.1 vs 37.5%) tended to be more commonly seen in the PHSC group than the PHS group. In the PHSC group, 13 patients underwent surgery, five of them were combined with TACE or RFA (radiofrequency ablation), four patients received interventional therapy and one patient underwent liver transplantation. 64.7% (11 of 17) of PHSC patients progressed or died between 1 and 16 months. In the PHS group, seven patients underwent surgery and seven received interventional therapy. 76.9% (10 of 13) of PHS patients progressed or died between 1 and 26 months.

**Table 1:** Clinical characteristics of the study patients with PHSC and PHS

Variable	PHSC (*n* = 23)	PHS (*n* = 16)	*P* value
Age (y)^a^	54.0 ± 9.9	54.4 ± 13.4	0.276
Male: female ratio	20:3	11:5	0.235
Liver hepatitis cirrhosis	14 (73.7)	10 (62.5)	0.716
Clinical manifestations	11 (52.4)	11 (73.3)	0.500
Tumor markers			
AFP	9 (47.4)	5 (33.0)	0.495
Ca19–9	5 (35.7)	2 (14.3)	0.385
CEA	1 (6.7)	0	0.999
Vascular invasion	13 (56.5)	4 (25.0)	0.099
Intrahepatic metastasis	9 (40.9)	4 (25.0)	0.490
Extrahepatic metastasis	12 (57.1)	6 (37.5)	0.325

Note. Unless otherwise specified, data are numbers of patients, with percentages in parentheses, *AFP* A-fetoprotein, *CA 19–9* Carbohydrate antigen 19–9, *CEA* Carcinoembryonic antigen

^a^Data are medians, with ranges in parentheses

### The morphologic features and accompanying findings of PHSC and PHS

Table 2 summarizes the morphologic characteristics and accompanying findings of PHSCs and PHSs. PHSCs and PHSs occurred more frequently from the subcapsular area in the right hepatic lobe (69.6% vs 75.0%) with a sharp margin. Tumors greater than 10 cm were more commonly seen in PHSs relative to PHSCs (*P* = 0.043). The PHSCs and PHSs mainly showed smooth (47.8% vs 37.5%) or bulging surface (43.5% vs 56.3%) and retraction of the capsule was rare. The capsule appearance occurred in around half of these patients and 54.5% (6 of 11) of capsule in PHSCs was incomplete. While there was no significant difference, hemorrhage was more common in PHSs than PHSCs (50.0% vs 26.1%).

**Table 2:** The morphologic features and dynamic enhancement characters of PHSC and PHS

Variable	PHSC (*n* = 23)	PHS (*n* = 16)	*P* value
Right lobe	16 (69.6)	12 (75.0)	0.999
Tumor diameter	72.6 ± 38.9	94.8 ± 44.4	0.823
≥10 cm	5 (21.7)	9 (56.3)	0.043
<10 cm	18 (78.3)	7 (43.8)	
Contour			0.999
Round	16 (69.6)	10 (62.5)	
Lobulated	2 (8.7)	2 (12.5)	
Irregular	5 (21.7)	4 (25)	
Margin			0.444
Sharp	19 (82.6)	11 (68.8)	
Indistinct	4 (17.4)	5 (31.3)	
Liver surface contour			0.786
Retraction	2 (8.7)	1 (6.3)	
Smooth	11 (47.8)	6 (37.5)	
Bulging	10 (43.5)	9 (56.3)	
Capsule appearance	11 (47.8)	6 (37.5)	*0.743*
Hemorrhage	6 (26.1)	8 (50.0)	0.179
Perfusion alteration	11 (47.8)	9 (52.2)	0.748
Cystic mass	4 (17.4)	8 (50.0)	0.041

Note. Data are numbers of lesions, with percentages in parentheses

### Comparison of enhancement characteristics of PHSC and PHS

**Fig. 1:**
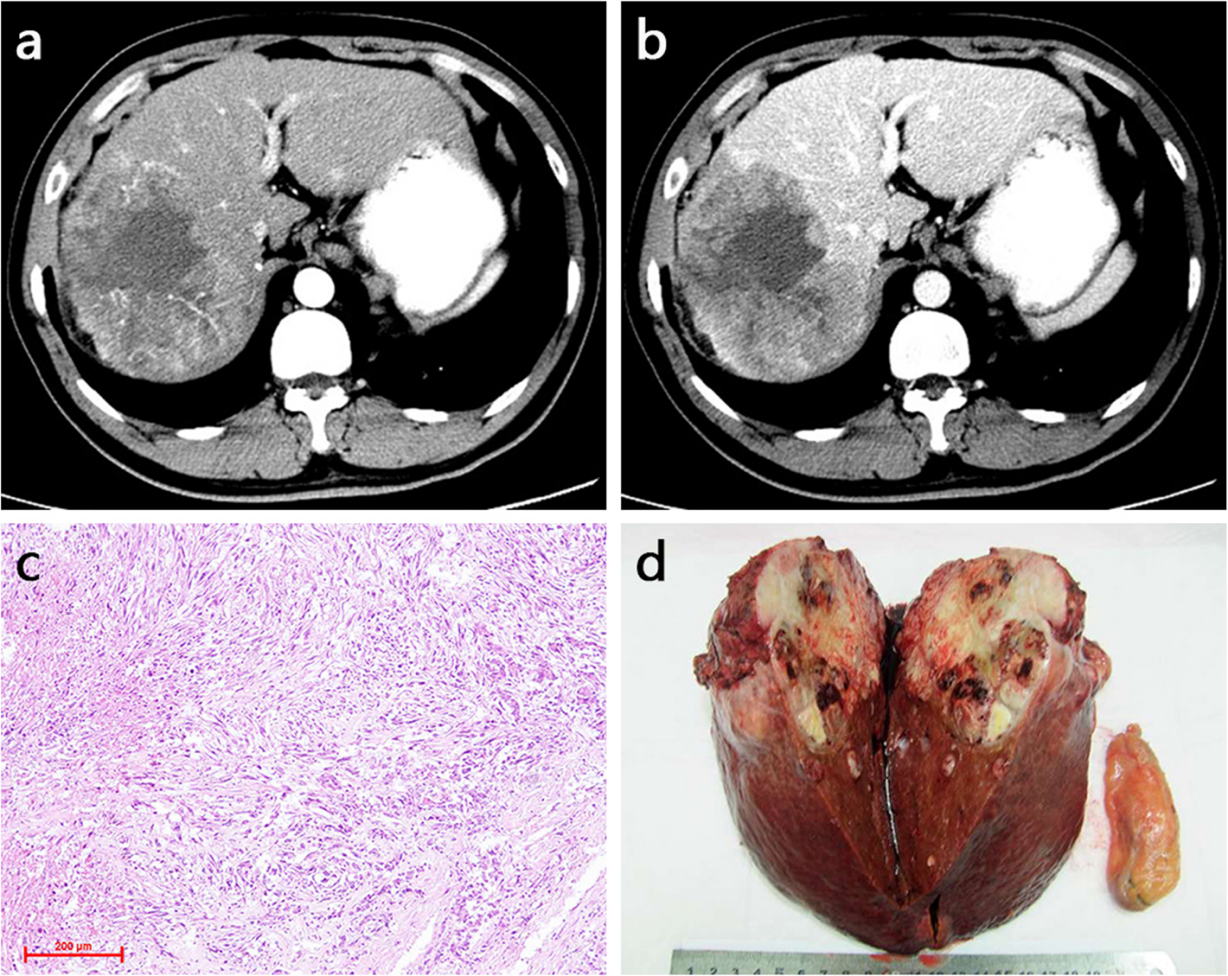
Sarcomatous hepatocellular carcinoma in a 32-year old man. The contrast-enhanced dynamic CT axial images exhibit the mass hyper peripheral enhancement on the AP (**a**) and washout on the PVP (**b**). H & E stain shows some neoplastic cells with pleomorphism(**c**). The bisected specimen displays a large solid tan mass with necrosis and a satellite lesion (**d**)

**Fig. 2:**
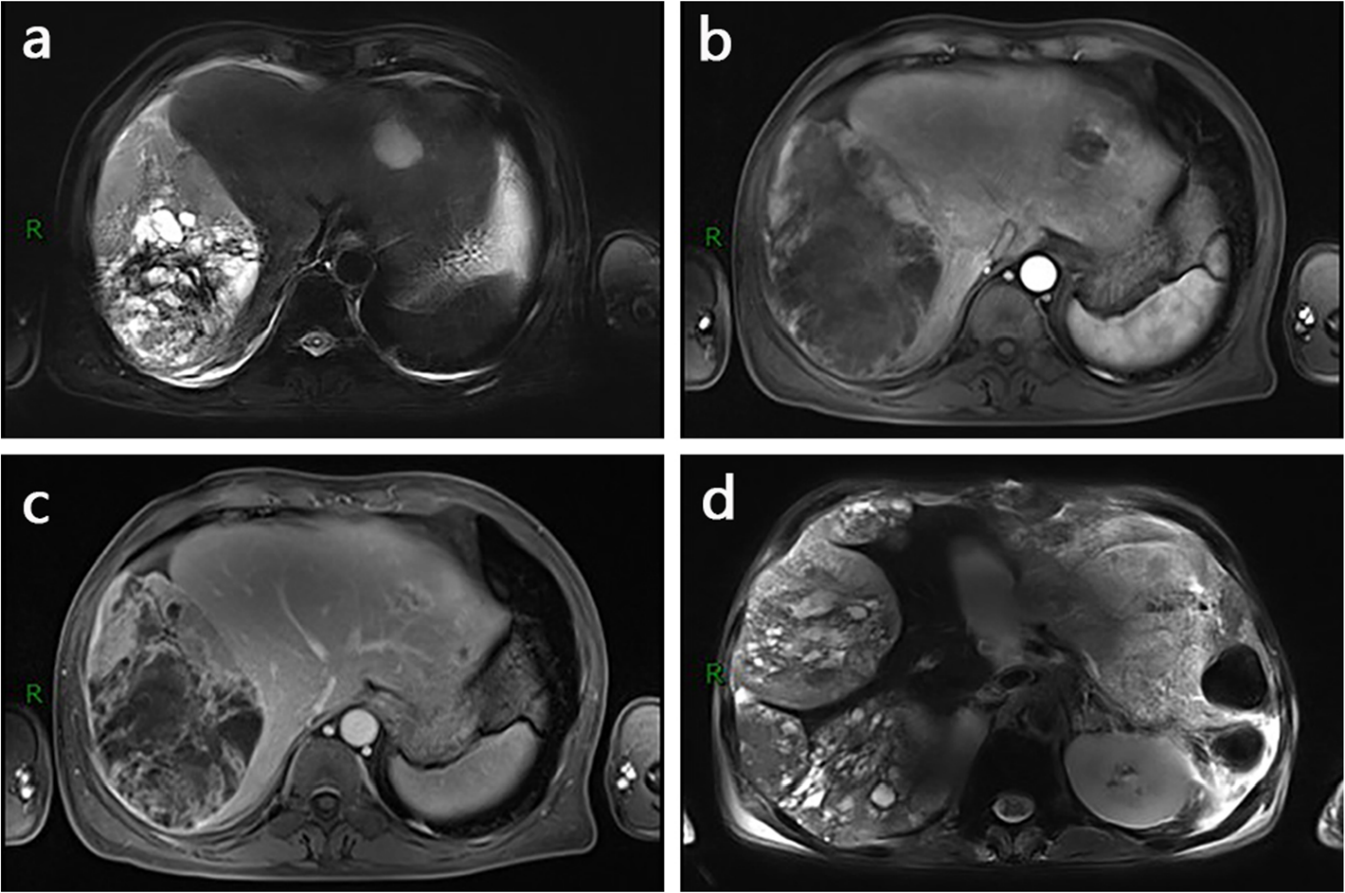
Sarcomatous intrahepatic cholangiocarcinoma in a 55-year old man. T2-weighted TSE BLADE sequence presents a protruding bulging mass with multilocular cyst-like changes and hemorrhage (**a**). Dynamic gadoxetic acid–enhanced MR images show hyper irregular peripheral enhancement on the AP (**b**) followed by peripheral wash out and centrally progressive enhancement with septa on the later phase (**c**). The mass recurs one month after surgery (**d**)

**Fig. 3:**
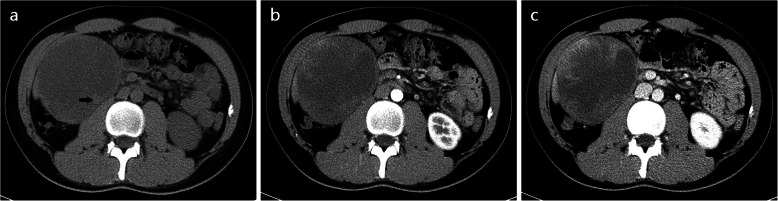
Hepatic undifferentiated embryonal sarcoma in a 22-year old man. Axial CT image shows a protruding bulging mass in hepatic segment VII with a large cyst-like area (**a**). Contrast-enhanced dynamic CT axial images present the mass hypo-enhanced peripherally on the AP (**b**) and centripetally in the PVP (**c**)

**Fig. 4:**
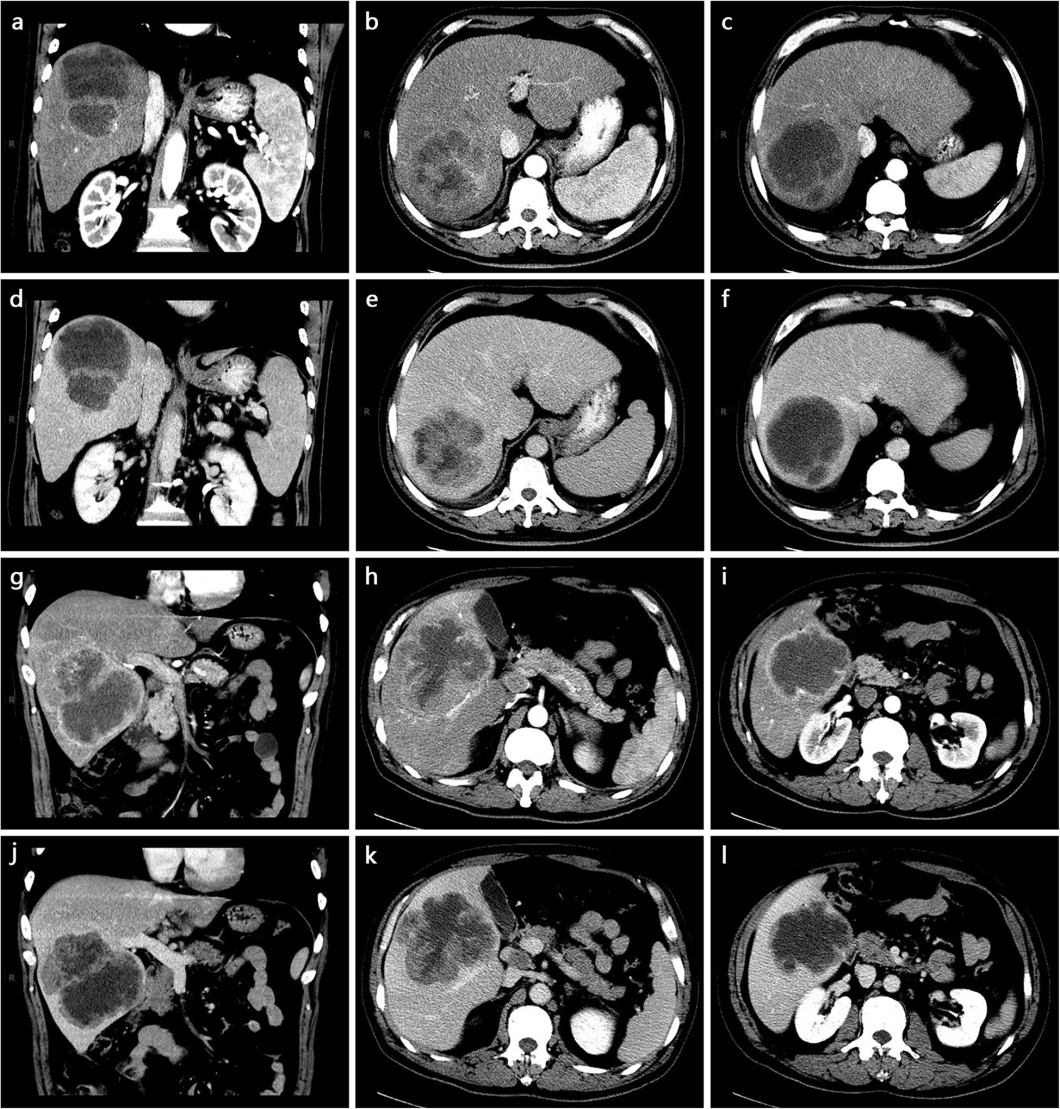
Hepatic leiomyosarcoma in a 62-year old man (**a-f**) and sarcomatous hepatocellular carcinoma in a 56-year old man (**g-l**). On the contrast-enhanced dynamic CT coronal and axial images, hepatic leiomyosarcoma (**a-c**) exhibits isoenhancement on the AP and persistent or progressive enhancement into the center on the later phase (**d-f**). The sarcomatous hepatocellular carcinoma presents obvious peripheral enhancement on the AP (**g-i**) and subsequent wash out (**j-l**)

Table 3 summarizes the enhancement characteristics of PHSC and PHS. There was significant difference in the AP enhancement (*P* = 0.027). Ring hyper-enhancement was more commonly seen in PHSCs than PHSs (60.1% vs 18.8%). Of the 14 patients in PHSCs with ring hyper enhancement on AP, 7 patients demonstrated rim enhancement and the others showed peripheral enhancement (Figs. 1, 2). The iso-hypo enhancement was more frequently detected in PHSs (Fig. 3) than PHSCs (56.3% vs 30.4%). A pattern of persistent or progressive enhancement was observed in both PHSCs and PHSs, and particularly in the PHSs, while wash out was more commonly seen in PHSCs (*p* = 0.017) (Fig. 4). For the vascularity of the whole tumor, PHSCs and PHSs were predominantly hypovascularity (78.3% vs 81.3%) and the cystic mass was more commonly seen in the PHSs than the PHSCs (50.0% vs 17.4%, *p* = 0.041). Notably, no or minimal enhancement with a nearly complete cystic appearance existed in PHSs in our study.

**Table 3:** The dynamic enhancement characters of PHSC and PHS

Variable	PHSC (*n* = 23)	PHS (*n* = 16)	*P* value
Vascularity^a^			0.999
Hypervascularity	5 (21.7)	3 (17.6)	
Hypovascularity	18 (78.3)	13 (81.3)	
AP enhancement			0.027
Ring hyperintensity	14 (60.1)	3 (18.8)	
Non-ring hyperintensity	2 (8.7)	4 (25.0)	
Iso- or hypointensity	7 (30.4)	9 (56.3)	
Dynamic pattern			0.017
Wash out	12 (52.2)	2 (12.5.0)	
Persistent or progressive	11 (47.8)	14 (87.5)	

Note. Data are numbers of lesions, with percentages in parentheses

^a^ The whole tumor was evaluated according the predominant parts more than 50%

## Discussion

In our study, we did not find any significant difference between the two tumors in the background of liver cirrhosis and the tumor markers such as AFP, CEA and CA1–99. Unlike previous reports that patients with PHSs had no evidence of hepatitis or cirrhosis [4, 18], ten (62.5%) of sixteen PHSs in our study were positive for hepatitis or cirrhosis. This percentage may be due to the situation of our particular infectious disease hospital, where a majority of people come with infectious diseases such as viral hepatitis. Fourteen (73.7%) of 19 PHSCs had a medical history of liver cirrhosis, similar to previous reports that hepatitis virus infection might have relationship with the occurrence of PHSCs [5, 19]. For PHSs, most of the laboratory tests came back negative [11, 15], but nearly half of the PHSC patients were positive for AFP, which might be helpful in its diagnosis.

Similar to previous studies [3, 12, 13, 20], the PHSCs and PHSs demonstrate hypovascularity probably for hemorrhage, necrosis, fibrous tissue or myxoid degeneration [21–24]. However, the AP enhancement and dynamic enhancement pattern were significantly different. The current study concluded that PHSCs mainly showed ring hyper-enhancement on the AP, followed a washout on the later phase. It was reported that the diverse tissue compositions of PHSC determine its enhancement pattern [6]. The PHSCs, especially the S-HCCs, were characterized by peripheral viable cancerous tissue (viable cells, higher microvascular densities and relatively less fibrous tissue) and central necrosis. The sarcomatous component comprises poorly differentiated cells that grow rapidly with the neovasculature unable to adequately supply the fast-growing malignant cells, resulting in necrosis. The PHSs generally present iso-or hypo enhancement on the AP and persistent or progressive enhancement on the later phase, similar to previous studies [12, 25]. The myxoid degeneration and the loose arrangement of the cells in PHSs could expand the extracellular space and the contrast agent in the extracellular space were accumulated gradually and expurgated slowly, leading to hypo-iso continuous or progressive enhancement.

Our study demonstrated the cystic mass was commonly seen in PHSs (*P* < 0.05). In our current study, some PHSs displayed nearly complete cyst-like masses with almost no enhancement simulating benign tumors, this was not seen in any PHSCs. There had been an emphasis on cystic-like appearance in PHSs, which was mainly attributed to the varying degrees of myxomatous change [11, 12, 14, 26, 27]. Hemorrhage also played a role in the cystic appearance, which was reported more frequently seen in PHSs than some other rare liver malignant tumors and attributed by rupture of the tumor for the serpiginous vessels [10, 11]. In previous studies, there was often extensive hemorrhage in PHS creating a huge cyst mass so that the underlying tumor was obscured and misdiagnosed as a hematoma, abscess or cystic tumor [28], which also occurred in our study. Although there was no statistical difference in the current study, hemorrhage was more commonly seen in PHSs than PHSCs (50.0% vs 26.1%). In addition, tumors larger than 10 cm in PHSs was detected significantly more frequently than in PHSCs (*P* < 0.05). It was reported that solid or cystic manifestations were different stages of PHSs and as the tumor grew, necrosis increased, tending to result in a cystic appearance. In summary, cystic lesions occurred more often in the PHSs and it might help us distinguish PHS from PHSCs.

The capsule invasion [19], vascular invasion or thrombosis, intrahepatic metastasis and lymph node metastasis were more prevalent in PHSCs and in our study the vascular invasion in PHSCs was close to significantly more common than the PHS (*P* = 0.099). The PHSCs were highly aggressive, and the presence of SC were considered to be closely related to the more invasive tumor biology, more common metastasis, low resectability and frequent postoperative recurrences [19, 29, 30]. By contrast, the PHSs usually involved the adjacent anatomic structures, and vascular invasion, metastases and lymph node involvement were less common [9, 28].

These tumors should also be differentiated from other liver masses [18]. The ring hyper-enhancement of PHSCs may mimic those of ICCs [31]. The elevated CA19–9 levels, bile duct dilation around the lesion and capsule retraction may be helpful for the differentiation of these lesions [32]. The global avid enhancement with washout and elevated AFP levels help us to differentiate the HCC from PHS and PHSC [18, 33, 34]. When the PHSs displaying as almost complete cyst-like mass as seen in our study, they should be distinguished from other cyst-like lesions such as hydatid cyst, abscess, biliary cyst or adenoma. It has been reported in studies that a cyst-like PHS could be frequently misdiagnosed as a hepatic cyst [4, 14, 26]. However, the presence of feeding vessels, the findings of hemorrhage and the abrupt increase in its size should alert us to the diagnosis of PHS [11, 28].

We should acknowledge several limitations to our study. First, for the retrospective study, it was technically unworkable to make a slice-by-slice imaging-pathology match. Second, the relatively small sample size had its intrinsic disadvantages; this, however, was inevitable for the rare incidence of the tumors. Third, there was no recognized international standard for the evaluation of the cystic tumors.

## Conclusions

The PHSC and PHS generally presented as a large subcapsular hypovascular mass. The ring hyperenhancement, wash out and more common vascular invasion favored the diagnosis of PHSC. The large mass with a diameter more than 10 cm, iso-hypo intensity/density on AP and pattern of persistent or progressive enhancement might alert us to the possibility of PHS. In spite of the presence of these meaningful diagnostic features, there were not specific for the diagnosis and differential diagnosis of PHSC and PHS. However, the absence of characteristic imaging manifestations of primary hepatic tumors should remind us of the possibility of these tumors.

## Data Availability

Original data and material are available from the corresponding author upon request.

## Abbreviations

PHSC: Primary hepatic sarcomatous carcinoma
S-ICC: Sarcomatous intrahepatic cholangiocarcinoma
S-HCC: Sarcomatous hepatocellular carcinoma
S-HCC–CC: Sarcomatous combined hepatocellular and cholangiocarcinoma
PHS: Primary hepatic sarcoma
UES: Undifferentiated embryonal sarcoma
LS: Leiomyosarcoma
SFT: Solitary fibrous tumor
HAP: Hepatic arterial phase
PVP: Portal venous phase
EP: Equilibrium phase
AFP: Alpha-fetoprotein
CA 19–9: Carbohydrate antigen 19–9
CEA: Carcinoembryonic antigen
CT: Computed tomography
MRI: Magnetic resonance imaging
